# A case of double appendix and cecum in an infant – case report

**DOI:** 10.1093/jscr/rjac125

**Published:** 2022-04-26

**Authors:** Murad Habib, Sadia Asmat Burki, Muhammad Amjad Chaudhary

**Affiliations:** Department of Paediatric Surgery, The Children's Hospital, Pakistan Institute of Medical Sciences, Islamabad, Pakistan; Department of Paediatric Surgery, The Children's Hospital, Pakistan Institute of Medical Sciences, Islamabad, Pakistan; Department of Paediatric Surgery, The Children's Hospital, Pakistan Institute of Medical Sciences, Islamabad, Pakistan

## Abstract

The duplication of the cecum and appendix is a rare congenital anomaly found incidentally on exploration for another indication. We present here a case of a female child at 18 months of life, who was a diagnosed case of anorectal malformation with a persistent cloaca and at the time of the surgery, duplication of the appendix and cecum was found. Both the appendices were attached to the cecum with a separate base. Thus, appendectomies and a sigmoid divided colostomy were performed.

## INTRODUCTION

The development of a double appendix is an extremely rare occurrence with an incidence of merely 0.004–0.009% [1]. It was in the year 1892 that the first case of the double appendix was ever clinically reported [2]. It is an anomaly that mostly presents in association with other congenital anomalies of the intestinal and genitourinary system [3]. The presence of the double appendix in a neonate has the potential to cause intestinal obstruction and it can even imitate the symptoms of intussusception and other abdominal pathologies [4]. With the congenital duplication of the appendix, there are reported cases of associated duplication of other structures like the colon [5] and other intestinal and genitourinary systems. The authors report a case of a newborn with persistent cloaca and double appendices at laparotomy, and discuss the pathogenesis of appendix duplication [6].

## CASE REPORT

A full-term child of 18 months was brought to Children’s hospital with complaints of inability to pass stool through the anal opening, abdominal distension and non-bilious vomiting. There was no family history of malformations, consanguinity and no history of medication to the mother during pregnancy. Physical examination revealed evidence of persistent cloaca as an abdominal distention and a single perineal orifice with partially fused labia and absence of an anal orifice. Routine complete blood count and biochemical tests revealed no abnormalities except a slight increase in white blood cells. All other milestones achieved. Radiographic examination revealed prominent fecal loaded gut loops as well as obscurity of mid-abdomen due to bowel gas shadows.

**Figure 1:**
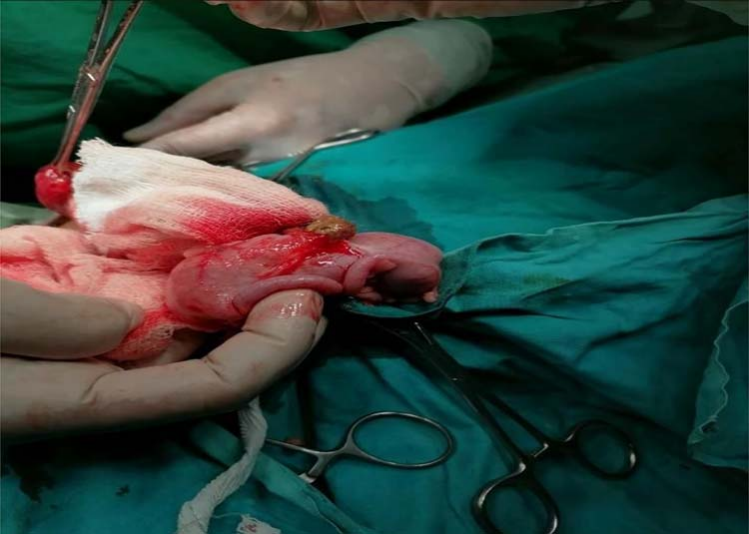
Double appendix shown as attached to the base of cecum.

A sigmoid divided colostomy was made through an oblique lower abdomen incision; a single opening in the vestibule, a free-floating cecum with two bases, a double appendix and a markedly distended distal pouch were found as shown in Fig. 1. The presence of a double appendix was the rare finding of this surgery that was meant to create temporary colostomies.

## DISCUSSION

**Figure 2:**
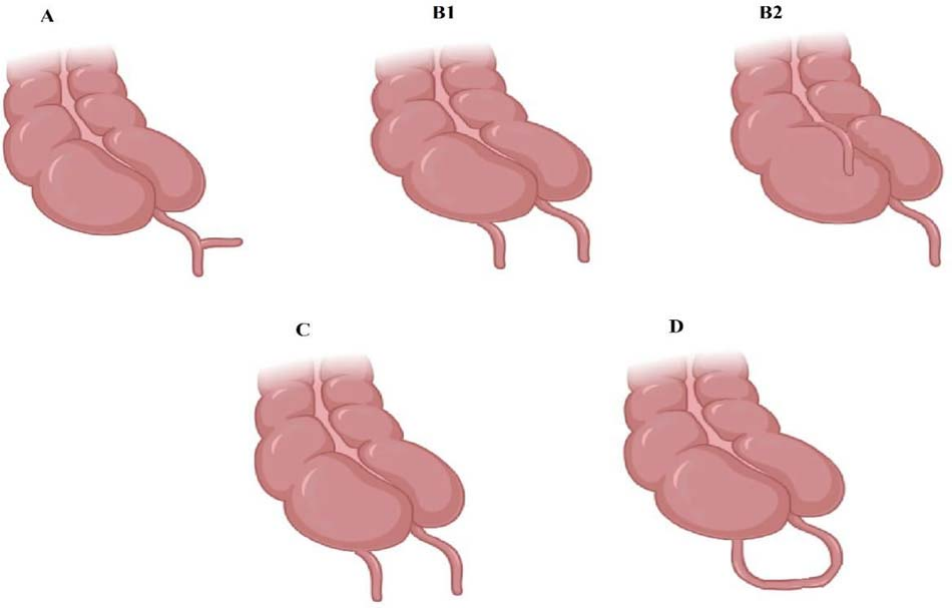
Type A shows partially duplicated appendix, Type B shows two discreet appendices, B1 bird beak like, B2 tenia coli, Type C has a duplicated cecum.

Congenital appendiceal anomalies are an infrequent occurrence. ‘Appendiceal Duplication’ and ‘Complete Appendiceal Absence’ are the two prevalent appendiceal congenital anomalies. These appendiceal anomalies remain mostly asymptomatic unless diagnosed as a part of diagnostic interventions for some other diseases. Symptoms appear mostly when the appendix or the appendices are obstructed and or inflamed. The clinical presentation can vary according to the location of the appendices [7]. The rarity of the development of a double cecal appendix can be known from the fact that only about 100 such cases have been reported since 1892 [8]. Duplication of the appendix is categorized, anatomically, by Wallbridge into various types, which are shown in Fig. 2.

**Table 1:** Cave-Wallbridge classification of duplication of appendix [8]

**Types**	**A**	**B1 (Bird-like)**	**B2 (Tenia coli variant)**	**C**	**D (Horseshoe appendix)**
No. of cecum	Solitary	Solitary	Solitary	Duplicated	Solitary
Appendiceal duplication	Incomplete	Duplicated	Duplicated	Duplicated	Duplicated
Development	Partial duplication of the appendix-like structure with one normal appendix is present at the base.	Due to failure of proper cloacal differentiation, two separate appendices originate from the singular cecum close to ileocecal valve.	The duplicated appendix is present along some colon tenia line; both the appendices originate from a single cecum.	Incomplete duplication of the hindgut results in two separate appendices originating from two separate cecum.	With a common duct, two appendices originate from a single cecum present in a relatively parallel fashion.
Related congenital anomalies	None	- Atresia of colon and anus. - External genitalia abnormalities. - Ectopic bladder. Small intestine and bladder communication.	None	- Duplication of various parts of hindgut, e.g. ileum, colon, anus, external genitalia and bladder, etc.	- An extremely rare type; sometimes, associated with other varying anomalies and sometime, without any other anomaly.

Type A: partially duplicated appendix of different extents with only a single cecum.

Type B: two discreet appendices with one cecum; this further has two more types. B1 (‘Bird-like’ Type) – two individual appendices present on both sides of the ileocecal valve. B2 (Tenia Coli Type) – an anatomically typical appendix along with the one originating along the line of tenia.

Type C: a duplicated cecum with each cecum having its separate appendix [9].

Type B1 and C are the ones that come with an increased incidence of other intestinal and genitourinary malformations and duplications, so if these types are encountered during the surgery special attention to other anatomical structures should be paid [10]. The Cave-Wallbridge classification is also modified in Table 1.

During the sixth week of embryological development, the cecal bud is the structure that lays down the foundation of the development of appendix. This cecal bud itself originates from the inferior end of the primary intestinal pouch. This cecal bud descends from its point of origin to give rise to many structures and it is during this process that appendix develops from its distal end as a slender diverticulum. It is anatomically placed posterior to the cecum and colon as it develops during the descent of the colon [11].

A case of triplication of the appendix has also been reported in a male child of 1 year with other corresponding congenital anomalies of the abdomen [12].

Duplication of the appendix is undoubtedly an infrequent anomaly; however, surgeons should always keep it in mind particularly in the instances when an appendix has been removed and the patient presents with the signs of symptoms of appendicitis or when the apparent appendix seems normal during the surgery, as it can have lethal repercussions for the patient and medico legal problems for the surgeon [13].

## CONCLUSION

Duplication of cecum and appendix is extremely rare but a case of anorectal malformation may present with it. Awareness of this condition and thorough intraoperative inspection is critical as not to miss underlying diagnosis and associated anomalies.

## CONFLICT OF INTEREST STATEMENT

There are no conflicts of interest.

## DECLARATION

This study was reviewed and approved by ethical review board committee of Pakistan Institute of Medical Sciences.

## PATIENT’S CONSENT

A written and informed consent was acquired from the guardian (father) regarding names and evidence used in this publication. And he had no objections what so ever.

## FINANCIAL SUPPORT

The author received no financial support for this article.
